# Tranexamic acid for treatment and prophylaxis of bleeding and hyperfibrinolysis

**DOI:** 10.1007/s00508-017-1194-y

**Published:** 2017-04-21

**Authors:** Ingrid Pabinger, Dietmar Fries, Herbert Schöchl, Werner Streif, Wolfgang Toller

**Affiliations:** 1 Clinical Department of Hematology and Hemostaseology, Medical University Vienna, Vienna, Austria; 2 Department of General and Surgical Intensive Care Medicine, Medical University Innsbruck, Innsbruck, Austria; 3 Department of Anesthesiology and Intensive Care Medicine, AUVA Accident Hospital Salzburg, Salzburg, Austria; 4 Academic Teaching Hospital, Paracelsus Private Medical University Salzburg, Salzburg, Austria; 5 Department of Children and Adolescents Medicine, Medical University Innsbruck, Innsbruck, Austria; 6 Department of Anesthesiology and Intensive Care Medicine, Medical University Graz, Graz, Austria

**Keywords:** Trauma, Surgery, Bleeding, Hyperfibrinolysis, Tranexamic acid

## Abstract

Uncontrolled massive bleeding with subsequent derangement of the coagulation system is a major challenge in the management of both surgical and seriously injured patients. Under physiological conditions activators and inhibitors of coagulation regulate the sensitive balance between clot formation and fibrinolysis. In some cases, excessive and diffuse bleeding is caused by systemic activation of fibrinolysis, i. e. hyperfibrinolysis (HF). Uncontrolled HF is associated with a high mortality. Polytrauma patients and those undergoing surgical procedures involving organs rich in plasminogen proactivators (e. g. liver, kidney, pancreas, uterus and prostate gland) are at a high risk for HF. Antifibrinolytics, such as tranexamic acid (TXA) are used for prophylaxis and treatment of bleeding caused by a local or generalized HF as well as other hemorrhagic conditions. TXA is a synthetic lysine analogue that has been available in Austria since 1966. TXA is of utmost importance in the prevention and treatment of traumatic and perioperative bleeding due to the resulting reduction in perioperative blood loss and blood transfusion requirements. The following article presents the different fields of application of TXA with particular respect to indications and dosages, based on a literature search and on current guidelines.

## Introduction

Massive bleeding after surgical interventions or severe trauma continues to be one of the most frequent life-threatening emergencies [1–3]. Trauma-associated hemorrhagic shock is the most frequent cause of avoidable deaths, with hyperfibrinolysis (HF) at the time of hospitalization having been identified as an independent predictor of mortality [4–6]. In addition, peripartum bleeding, with a proportion of up to 25% of pregnancy-associated complications, ranks first along with thromboembolism, among the causes of maternal death [7].

Hemostatic disorders in the context of massive bleeding have long been deemed as coagulopathies resulting from blood loss, dilution and consumption, forming the “lethal triad” along with hypothermia and acidosis. More recent studies have shown, however, that shock and the resulting hypoperfusion may trigger coagulopathy independently of tissue trauma, with HF being regarded as the underlying mechanism [8].

Since the 1960s the non-specific serine protease inhibitor aprotinin and the inhibitors of plasminogen activation tranexamic acid (TXA) and/or ε‑aminocapronic acid (ΕΑΧΑ) have been used in the treatment of HF. Aprotinin was, however, withdrawn from the market in 2007, since massive side effects had been observed in the course of complex cardiovascular surgery [9]. First described some 50 years ago, TXA has been widely used for the prevention and therapy of hemorrhages and/or primary and secondary HF. In recent years interest has increasingly focused on intravenous administration, since it has been shown that early administration of TXA after severe trauma can significantly increase the rate of survival [10–13]. Accordingly, in 2011 the World Health Organization (WHO) added TXA to its list of essential medicines.^1^ In addition, the reduction of perioperative blood loss and the resulting decline in the demand for transfusions have induced European societies (Task Force for Advanced Bleeding Care in Trauma and the European Society of Anesthesiology) to publish in recent guidelines a 1A recommendation for the use of TXA in the case of traumatic and perioperative bleeding [2, 3].

This article presents the different fields of application of TXA, including those outside anesthesia and intensive care medicine, on the basis of current studies and guideline recommendations regarding indications and dosage schedules.

## Hyperfibrinolysis

### Pathophysiology

By definition, HF is a state of increased clot resolution that may be associated with severe, potentially life-threatening hemorrhage. It may be caused by excessive plasmin formation or a reduction of plasmin decomposition due to a depletion of α_2_-antiplasmin. Plasmin is capable of cleaving both fibrin and fibrinogen. The resulting fibrin cleavage products, which inhibit the cross-linking of fibrin, can aggravate this effect [14].

### Disorders associated with HF

Clinical studies have shown that a number of pathological conditions may go hand in hand with the activation of the fibrinolytic system. In recent years the focus has increasingly been on severe trauma with subsequent tissue hypoxia. HF has also been observed in the context of severe postpartum hemorrhage [15]. Furthermore, an increase in the activation of fibrinolysis has been reported in the course of extracorporeal circulation and in the case of liver transplantations [16–18].

### Diagnosis

Diagnosing HF often proves difficult, since no specific tests that would allow prompt therapeutic decisions are currently available. Tests such as the determination of plasmin-antiplasmin complexes or α_2_-antiplasmin assays are time-consuming and hardly practicable in everyday clinical work. The same holds for the euglobulin lysis time, long seen as the gold standard but both time-consuming and fault-prone [19]. Viscoelastic tests such as thromboelastometry or thrombelastography (ROTEM and TEG, respectively), on the other hand, can detect HF but only if plasmin-antiplasmin levels are high or α_2_-antiplasmin levels are very low [20]. There is an urgent need for better and more sensitive diagnostic analyses and rapid HF monitoring to facilitate differentiation between hyperfibrinolytic states and other coagulopathies [21]. Moreover, it would be important to be able to identify patients who could be treated with TXA, since recent data suggest that not all patients benefit from TXA therapy [22, 23].

## Mode of action of tranexamic acid

TXA is a synthetic lysine analogue that inhibits conversion of plasminogen to plasmin by preventing plasminogen from binding to the fibrin molecule. TXA also inhibits plasmin activity directly, although only at higher doses [24]. TXA inhibits fibrin cleavage, thus reducing the risk of hemorrhage. It also blocks binding of α_2_-antiplasmin and inhibits inflammatory reactions. Compared with epsilon‐aminocaproic acid (EACA), TXA is more potent by a factor of 10 [25]. The substance can be administered orally or intravenously (Table 1), its oral bioavailability ranging from 30–50%. With a plasma protein binding of 3% it can completely cross the placenta. Metabolism of TXA in the liver is low, renal clearance amounts to 95% [26], and the half-life in adults is approximately 2.3 h [27].

**Table 1:** Therapeutic indications for tranexamic acid [27]

**Intravenous administration**
Prophylaxis and treatment of bleeding due to a local or systemic hyperfibrinolysis in adults and children over the age of 1 year
*Bleeding in which hyperfibrinolysis is considered to be involved:* Menorrhagia and metrorrhagia Gastrointestinal bleeding Bleeding in urinary tract infections, postoperative bleeding following prostate or urinary tract surgery
Ears, nose and throat (ENT) surgery (adenoidectomy, tonsillectomy, dental extractions)
Gynecological surgery or obstetric hemorrhage
Abdominal and thoracic surgery and other major surgery, e. g. cardiac surgery
As antidote in bleeding requiring immediate treatment while on fibrinolytic treatment
**Oral administration**
Hypermenorrhea (menorrhagia)
Prostatectomy
Epistaxis
Conisation of the cervix
Prophylaxis of recurrent bleeding in traumatic hyphema
Dental extraction and other interventions in ENT area in patients with hereditary coagulopathies
Mucosal bleeding in patients with coagulopathies
Hereditary angioneurotic edema

So far there have been no reports suggesting the presence of serious side effects, not even with high dosages and long-term administration (Table 2; [28]). Since renal insufficiency carries the risk of accumulation of TXA, administration of TXA is contraindicated in patients with severe kidney dysfunction (Table 3). Dosages need not be modified in patients with impaired liver function and elderly patients with no kidney dysfunction (Table 4). In patients with slight to moderate kidney dysfunction TXA dosages should be reduced in dependence on serum creatinine levels (for details see SmPC Cyklokapron® [27]).

**Table 2:** Clinically relevant adverse effects of tranexamic acid (expert opinions) [27]

Gastrointestinal disturbances (nausea, vomiting, diarrhea)
Drop of blood pressure/dizziness following a too fast intravenous administration
Incidental allergic skin reactions
Infrequent temporal vision impairment
Convulsions

**Table 3:** Contraindications of tranexamic acid [27]

Hypersensitivity to TXA
Early pregnancy, in late pregnancy only when vitally indicated
Disturbances of color vision
Massive bleeding in the upper urinary tract (risk of ureter obstruction due to clot)
Acute venous or arterial thrombosis
Severe renal impairment
History of convulsions
Intrathecal and intraventricular injection, intracerebral administration (risk of cerebral edema and convulsions)
Diseminated intravascular coagulation (DIC) without severe hemorrhage

**Table 4:** Dosage and administration of tranexamic acid

According to the SmPC the following dosage guidelines apply to adults [27]:
*1. Oral administration (1 tablet = 0.5 g).* The recommended standard dose is 2–3 times daily 2–3 tablets (1–1.5 g), daily dosage 2–4.5 g
*2. Intravenous administration (1 ampoule = 5 ml = 0.5 g) in fibrinolysis:* The recommended standard dose is 2–3 times daily 0.5–1 g (1–2 ampoules à 5 ml) by slow intravenous injection (1 ml/min)
*3. Intravenous administration in general fibrinolysis:* The recommended standard dose is 1 g (2 ampoules à 5 ml) every 6–8 h by slow intravenous injection (1 ml/min), corresponding to 15 mg/kg body weight

## Tranexamic acid in acute hemorrhagic events

### Trauma patients

**Fig. 1:**
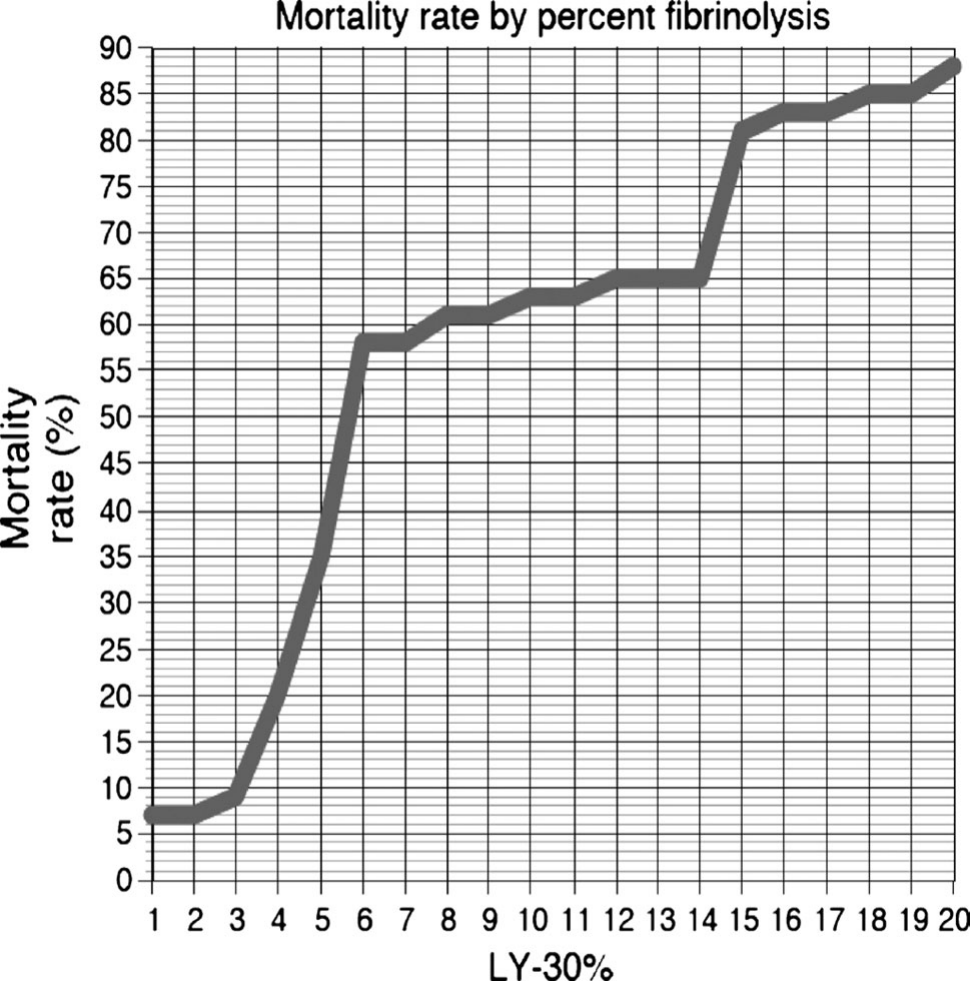
Mortality rate by percent fibrinolysis. Rate of amplitude reduction 30 minutes after the maximum amplitude (LY30) is reached and its correlation to mortality. HF was defined as more than 7.5% amplitude reduction 30 minutes after maximal amplitude (LY30). At an LY30 of 3% or less, 30-day mortality was 9%. However, once the LY30 extended to more than 3%, mortality increased to 20%. Large increases in mortality were also seen at LY30’s of greater than 4% (35%), greater than 5% (58%), and greater than 15% (81%). Reproduced with permission from [6]

Unlike elective surgery patients, 25–35% of all patients with severe physical injuries already show some form of coagulopathy when admitted to the shock room. As compared with patients who have been treated for deranged coagulation, these early coagulopathies are associated with a four-fold higher mortality [29]. Profibrinolytic activation appears to play a decisive role in these cases. The extent of posttraumatic HF apparently greatly depends on the extent of shock and the resulting hypoxia and tissue injury. The more pronounced the shock, the more severe HF will be (Fig. 1) [8]. The risk of mortality increases with the severity of HF. Several studies revealed that HF exceeding 3% was associated with a dramatic increase in mortality [5, 6, 30–32].

The CRASH-2 study published in Lancet in 2010, which comprised 20,211 patients, showed that the use of TXA (loading dose of 1 g TXA for 10 min + 1 g infusion over 8 h) compared to matching placebo resulted in a significant reduction of overall (14.5% vs. 16%, respectively) and hemorrhage-induced (4.9% vs. 5.6%, respectively) mortality in trauma patients without increasing the rate of thromboembolism [10]; however, the CRASH-2 data should be viewed with caution as it was performed mostly in developing and threshold countries and no influence on transfusion rates by TXA was documented. The CRASH-2 subgroup analyses have shown that all patient groups (severe shock, traumatic brain injury) benefited from TXA administration, no differences being revealed between penetrating and blunt trauma. The CRASH-2 study also did not show any difference with regard to thromboembolic events; however, a post hoc analysis showed that administration of TXA later than 3h after the initial trauma was associated with an increase in mortality [33], which suggests that TXA should be administered as early as possible [2].

**Fig. 2:**
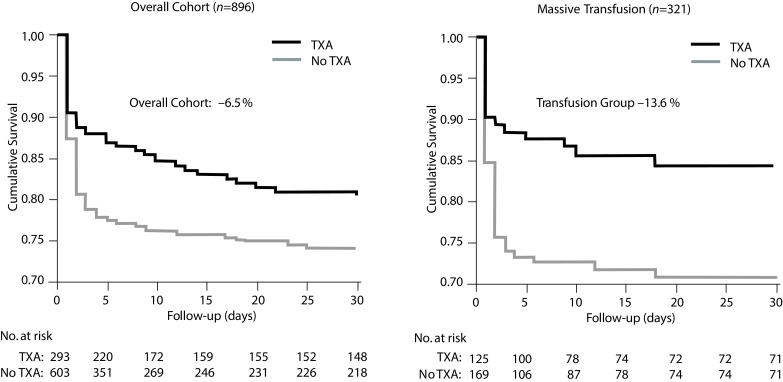
Cumulative survival of military TXA administration in the overall cohort and in patients with massive transfusion from the Trauma Emergency Resuscitation (MATTERs) Study. (Data from Morrison et al. [12])

A retrospective study by Morrison et al. of severely injured soldiers (*n* = 896; TXA: *n* = 293) demonstrated convincingly that administration of TXA as compared to cryoprecipitate resulted in a clearly lower mortality in the group of patients treated with TXA (massively transfused: –13.6%; total population: –6.5%; Fig. 2) [12]. In the group of patients who received massive transfusion TXA was independently associated with less coagulopathy (*p* = 0.003) and survival (OR = 7.23) with a relative risk reduction of mortality of 49% compared to 27% in the overall TXA cohort. On the other hand, the rate of thromboembolic events was higher in the TXA group.

In a subsequent study (*n* = 1332) it was shown that patients exclusively treated with cryoprecipitates (*n* = 168) exhibited the same survival rates as patients treated only with TXA (*n* = 148); the survival rate was highest in patients administered both TXA and cryoprecipitate (*n* = 258) [13].

In line with the recommendations of the S3 guidelines on treatment of polytrauma/severely injured patients of the German Society of Accident Surgery (Deutsche Gesellschaft für Unfallchirurgie, DGU) [34] an antifibrinolytic agent (e. g. 2 g TXA) should be administered whenever HF is suspected. It is recommended to embed administration of the antifibrinolytic agent in an overall therapy plan for treating coagulopathy, since in the course of HF the consumption of fibrinogen may frequently increase to such an extent that complete defibrination results. This fibrinogen depletion has to be compensated for once HF has been overcome [35] or in other words, the antifibrinolytic agent should be applied before fibrinogen is administered whenever HF is suspected [36]. For polytrauma patients the Austrian Society for Anaesthesiology, Resuscitation and Intensive Care (ÖGARI) and European guidelines [2, 3] recommend early administration of TXA (in the shock room at the latest). The recommended dosages are:

- 1 g for 10 min, followed by 1 g over 8 h [2] or
- 20–25 mg/kg body weight (BW) followed by continuous administration (1–2 mg/kg BW/h) [3]
- Special care should be taken in the case of intensive care patients in the presence of septicemia or disseminated intravascular coagulopathy (recommendation grade A)^2^ [37]

Rapid bolus administration of TXA might result in a further drop in blood pressure in shock patients and should be avoided [23].

### Postpartum hemorrhage

Postpartum hemorrhage (PPH) is among the leading causes of maternal mortality worldwide [38, 39]. In a series of controlled studies on PPH in vaginal deliveries TXA proved capable of reducing the extent of peripartum bleeding and time to hemostasis [40–43]. Similarly, administration of TXA in the course of cesarean sections resulted in a reduction of bleeding time and blood loss and, in some studies, of transfusion needs (Table 5; [44–54]).

**Table 5:** PPH: results of clinical studies with tranexamic acid in obstetrics

Authors	*N*	Method	Results
*Vaginal delivery*			
Dulcoy-Bouthers et al. [40]	144	RCT, double-blind 4 g TXA + 1 g/h in 6 h = 10 g	Bleeding duration ↓ Progression to severe PPH (>800 ml) ↓
Yang et al. [41]	400	RCT (prospective, double-blind) 0.5 g vs 1.0 g TXA vs. PAMBA*	Total blood loss ↓
Gungorduk et al. [42]	454	RCT TXA 1.0 g vs. placebo	Blood loss ↓ Progression to severe PPH (>500 ml) ↓
Bouet et al. [43]	289	Retrospective single centre analysis of policy before and after use of high dose TXA 10 g	No difference in blood loss
*Cesarean delivery*			
Goswami et al. [44]	90	RCT (prospective, double-blind) 10 mg/kg TXA in 20 min, then 10 vs. 15 mg/kg TXA vs placebo	Total blood loss in both TXA groups ↓ significantly less with 15 mg/kg
Gai et al. [45]	180	RCT (multi centre, prospective, randomized controlled) 1 g TXA over 5 min, 10 min before CS vs not	Blood loss until 2 h after delivery ↓
Abdel Aleem et al. [46]	740	RCT (Single centre, open, controlled) 1 g TXA over 10 min, 10 min before CS vs. not	Blood loss until 2 h after delivery ↓ Hemoglobin decline ↓
Sekhavat et al. [47]	90	RCT (prospective, randomized, double-blind) 1 g TXA over 10 min, 10 min before CS vs. not	Blood loss until 2 h after delivery ↓ Hemoglobin decline ↓
Halder et al. [48]	100	Case-control study TXA before CS vs not	Blood loss until 2 days postpartum ↓ Hemoglobin decline ↓
Movafegh et al. [49]	100	RCT (prospective, double-blind, randomized, controlled) 10 mg/kg TXA vs placebo	Blood loss until 2 h postpartum ↓ Hemoglobin decline ↓
Sentürk et al. [50]	223	RCT (prospective, double-blind, placebo controlled) 10 mg/kg for 5 min, 10 min before CS vs placebo	Intra- and postoperative blood loss ↓
Xu et al. [51]	174	Randomized case-control study 10 mg/kg TXA before CS or not	Blood loss until 2 h postpartum ↓ Progression to PPH ↓
Gohel et al. [52]	100	Randomized, prospective case-control study 1 g TXA over 5 min	Blood loss until 2 h postpartum ↓
Shahid et al. [53]	74	RCT (prospective, randomized, double-blind) 1 g TXA	Intraoperative blood loss ↓
Gungorduk et al. [54]	660	RCT (prospective, double-blind, placebo controlled) 1 g TXA	Intraoperative blood loss ↓

↓ = difference to placebo significant

*CS* cesarean section, *RCT* randomized clinical trial, *PPH* postpartum hemorrhage, *PAMBA* para-aminomethylbenzoic acid, *TXA* tranexamic acid

According to a Cochrane analysis (2010) of two randomized, controlled studies administration of 0.5 g and 1 g, respectively, of TXA reduced both blood loss and transfusion needs after vaginal births and cesarean sections [55]. A systematic review (1760 births) also found that the administration of TXA as compared with placebo resulted in a significant reduction of blood loss and the frequency of allogenic transfusion (RR 0.34, 95%CI 0.2–0.6) [56]. This finding is supported by a recent meta-analysis (2531 births) comprising 11 studies (Table 7; RR 0.23, 95%CI 0.10–0.57, *P <* 0.01) [57].

Accordingly, the European Society of Anaesthesiology (ESA) recommends administration of TXA in the case of peripartum and postpartum hemorrhage in order to reduce the extent of blood loss, the duration of bleeding and the need for allogeneic blood products (1B) [3]. Moreover, there is a 2C recommendation for the administration of TXA prior to cesarean section. In the case of antepartum hemorrhage TXA administration may be taken into consideration (2B) [3].

An international interdisciplinary expert consensus recommends for the treatment of postpartum hemorrhage the administration of 2 g TXA i. v. prior to supplementing fibrinogen, with dosages ranging from 1 to 3 g [58].

### Menometrorrhagia

Pathologically excessive and/or prolonged uterine bleeding is one of the most frequent symptoms in women suffering from coagulation abnormalities [59]. The prevalence of menorrhagia in patients suffering from von Willebrand disease is 32–100% (platelet dysfunction: 5–98%; rare factor deficiency states 35–70%) [60–62].

According to various studies TXA administered to menorrhagic women resulted in a significant reduction of menstrual blood loss while not increasing the risk of thrombosis [63] and is successfully used to treat menorrhagia associated with a number of coagulopathies [64, 65]. In patients with hypermenorrhoea and/or menorrhagia TXA (administered from the 1st to the 5th day of the cycle) can reduce blood loss by 35–60% [60, 66].

The recommended dose is 2–3 tablets TXA (1–1.5 g) 3–4 times a day for 3–4 days (starting immediately after onset of heavy bleeding). In the case of excessive bleeding the dose may be increased but should not exceed a maximum daily dose of 4 g [27].

### Mucocutaneous bleeding with coagulopathies

Antifibrinolytics are also used for many hemorrhagic conditions, and especially for mucosal bleeding of the nose or gums. Mouthwashes with antifibrinolytics are useful to prevent bleeding after tooth extractions [67, 68]. In many cases of mild to moderate mucosal bleeding topical and systemic administration of antifibrinolytics combined with topical hemostasis will be sufficient [69].

In patients suffering from hemophilia, von Willebrand disease or congenital platelet dysfunctions, topical^3^ (in the form of mouthwashes), buccal or intravenous administration of TXA will help reduce hemorrhage and blood loss, particularly mucosal bleeding. In cases of moderate and mild hemophilia, von Willebrand disease type 1 (mild deficiency) and storage pool disease (thrombocyte granular defect) it is recommended to use desmopressin jointly with TXA [62, 70, 71]. On the other hand, combined administration of activated factor concentrate (Anti-Inhibitor Coagulant Complex, FEIBA/Baxter) and TXA is not recommended in view of the rather unpredictable increase in coagulability (hypercoagulability). In hemorrhage of the upper urinary tract TXA may favour the formation of obstructive clots in the bladder, hence it is not recommended either.

### Gastrointestinal bleeding

In acute bleeding in the upper gastrointestinal tract TXA reduces mortality [3, 72, 73], with a recent Cochrane analysis reporting a relative mortality risk of 0.60 and a relative after-bleeding risk of 0.72 [74]. This review comprised eight randomized controlled studies published between 1973 and 2011; however, the authors *do not* recommend routine use of TXA in the case of gastrointestinal bleeding (“tranexamic acid cannot be recommended for routine clinical practice”), pointing to a current HALT-IT study for which a total of 8000 patients with gastrointestinal bleeding are to be recruited. To date (November 2016) more than 5670 persons have been included.^4^

## Tranexamic acid in elective applications

### Orthopedic surgery

Administration of antifibrinolytics in the course of major orthopedic surgery (total hip or knee replacement) is associated with a reduction of perioperative blood loss and allogeneic blood transfusions [75–78]. A comprehensive retrospective cohort study (*n* = 872,416) has furthermore shown that TXA results in a significant reduction of the probability of transfusion in patients undergoing total hip or knee replacement (OR 0.31–0.38; *p* < 0.001) without increasing the risk of thromboembolism, kidney failure or combined complications [79]. This is also confirmed by a meta-analysis of 46 randomized controlled studies on 2925 orthopedic surgery patients, which also pointed to a reduction of total intraoperative and postoperative blood loss [80].

In patients undergoing total knee replacement without blood arrest, blood transfusions could be completely dispensed with, when TXA was administered twice (preoperatively and postoperatively i. v. 15 mg/kg BW), while 32% of patients in the control group required transfusions. In addition, postoperative drainage blood loss was significantly lower vs. placebo, and the hematocrit and hemoglobin values of the verum group were higher [81]. In patients undergoing total knee replacement, oral TXA (1 g preoperatively followed by 1 g every 6 h over a period of 18 h postoperatively) proved effective in reducing postoperative fibrinolysis [82, 83]. In hip surgery TXA (15 mg/kg BW as either a single or double bolus at the start of the intervention and 3 h thereafter) resulted in a reduction of allogeneic blood transfusions but increased the risk of hypercoagulability [84–86].

The ESA recommends the use of TXA in the case of total hip endoprostheses (TEP), knee joint replacement and major spinal surgery (2A) [3]. It should be pointed out, however, that TXA may cause hypercoagulability in some patient groups (pre-existing thromboembolic events, hip fracture/cancer surgery, age > 60 years, women). It is therefore recommended to perform an individual risk-benefit analysis rather than administer TXA routinely (2A).

### Cardiovascular surgery/coronary artery bypass graft (CAGB) interventions

Numerous studies show that the use of antifibrinolytic agents in the course of cardiovascular operations reduces blood loss; it seems that TXA is superior to EACA with regard to perioperative transfusions [87]. Since the withdrawal of aprotinin from the market, at the latest, TXA has been the standard antifibrinolytic treatment in heart surgery [88]. The two guidelines for anticoagulation management published by the Society of Thoracic Surgeons (STS) and the European Association for Cardio-Thoracic Surgery (EACTS) deal with the intraoperative use of antifibrinolytics in heart surgery: both TXA and EACA are consistently recommended to minimise blood loss and transfusion needs [1, 89]. In a double-blind, randomized, placebo controlled trial (*n* = 222) TXA (preoperative bolus followed by intraoperative permanent infusion) reduced drainage volume and transfusion needs in elective coronary artery bypass graft (CABG) [90]. Similar results were obtained in a meta-analysis of 25 RCTs (*n* = 5411) and large-scale observational studies (*n* = 5977), which confirmed the efficacy of TXA, as compared with placebo, in reducing blood loss, allogeneic blood transfusion needs and the reoperation rate due to postoperative hemorrhage [91].

The ESA guidelines recommend the use of TXA prior to CABG interventions (1A); likewise, intraoperative administration of TXA should be considered in order to reduce perioperative bleeding in the course of high, medium and low-risk cardiovascular surgery (1A). Also, topical (see footnote 3) application of TXA in the thoracic cavity is recommended to reduce postoperative blood loss after CABG (1C).

A current dose comparison study on patients undergoing heart surgery while connected to a heart-lung machine revealed, up to day 7, a higher efficacy in terms of blood loss, lower incidence of transfusions and reoperation rate in the high-dose group (TXA 30 mg/kg BW bolus + 16 mg/kg BW/h) as compared with the low-dose group (TXA 10 mg/kg BW bolus + 1 mg/kg BW/h), while reporting no difference with regard to the incidence of blood product transfusion; in other words, the percentage of patients that required blood transfusions up to 7 days after the intervention was not reduced [92].

TXA dosages in cardiac surgery with cardio-pulmonary bypass (CPB) currently relate mostly to those used in the BART Study [9]: TXA bolus 30 mg/kg BW prior to CPB, followed by continuous infusion with 16 mg/kg BW/h up to the end of the operation [9]. It should be noted that open heart surgery in particular carries a higher risk of seizures caused by even moderate doses of TXA (24 mg/kg BW/day) [84].

In one Austrian centre (Graz) the incidence of seizures was successfully reduced by lowering, on a trial basis, the TXA dose to one half of the dose used in the BART study (bolus for all patients 1 g [= 10 ml] prior to HLM; immediately thereafter infusion of 8 mg/kg BW/h up to the end of surgery): While the efficacy was slightly attenuated, virtually no seizures were observed (W. Toller, personal communication).

### Liver surgery

#### Orthotopic liver transplantation

In orthotopic liver transplantations (OLT) antifibrinolytic therapy reduces both blood loss and transfusion needs (B) [3]. A meta-analysis has shown that both TXA and aprotinin reduce the need for erythrocyte concentrates [93]. Moreover, a Cochrane review concluded that antifibrinolytic therapy helps reduce blood loss and perioperative transfusion needs [94], with TXA and EACA being equally effective.

Treatment of hyperfibrinolysis (confirmed by the presence of microvascular blood oozing and/or thrombelastography or rotation thromboelastometry, TEG/ROTEM measurement) with antifibrinolytics is recommended; lowest effective doses are uncertain, TXA being currently administered in gradually increasing dosages of 1–2 g [3].

#### Liver resection

In the course of liver resections for hepatocellular carcinoma TXA reduced average total blood loss and transfusion needs significantly as compared with placebo [95]. A Cochrane review also reported a reduction of allogeneic blood transfusions in patients receiving TXA or aprotinin [96].

According to ESA recommendations [3] antifibrinolytic therapy should be considered in patients with cirrhosis of the liver and liver resection (2C).

### Prostate surgery

Intraoperative blood loss during prostate surgery is significantly lower with TXA than with placebo [97]. This was also confirmed by smaller-scale studies on prostatectomy with oral TXA (post-op 1.5–3 g/day for 3 days) [98, 99].

After initial intravenous therapy during the first three postoperative days, 2–3 tablets (1–1.5 g) may be given twice or three times daily for 7 days or until hematuria can no longer be detected macroscopically [27].

### Conisation and gynecologic oncologic surgery

TXA reduces both the incidence of secondary hemorrhage after cervical conisation [100] (B) and the incidence of perioperative bleeding during gynecologic oncologic surgery [101] (C) [3].

According to the prescribing information the dosage of TXA after cervical conisation should be 3 tablets (1.5 g) three times a day for 12–14 days post operatively [25]; however, administration of TXA is *not* recommended for benign gynecological interventions, such as myomectomies (2B) [3, 102].

### Pediatrics and pediatric surgery

In pediatrics TXA has a wide field of application (Table 6). It is used to prevent and treat hemorrhage and to combat hyperfibrinolysis; however, literature data on the use in children is greatly limited.

**Table 6:** Recognized indications of tranexamic acid in pediatrics (expert opinion)

Excessive fibrinolysis (e. g. liver transplantation, medication induced)
Adjuvant as hemostatic agent, in hemophilia and von Willebrand disease (e. g. dentistry, not in renal bleeding!)
Mucosal bleeding (topical^a^, oral, intravenous), exception: bleeding of the upper urinary tract
Primary and adjuvant in hereditary thrombopathies/thrombopenia [102, 103]
PAI 1 deficiency, α_2_-plasmin inhibitor deficiency, hereditary telangiectasia
*Adjuvant in:* acquired thrombocytopenia cardiac surgery bypass surgery

^a^Topical use: TXA (solution for injection) is locally applied without dilution or diluted with NaCl 0.9%; when applied in the mouth the swallowed amount should be added to the total dosage. The topical application is not included in the SmPC

In children undergoing cardiac surgery or surgery for scoliosis with a high bleeding risk, TXA has significantly reduced perioperative blood loss and the need for erythrocyte concentrates [103, 104]. Similar effects have also been reported in the context of pediatric craniosynostosis operations [105].

Perioperative antifibrinolytic therapy to reduce blood loss and transfusion needs is proposed for both heart surgery and non-cardiac surgical interventions (2A) [3].

Optimum dosages for pediatric patients have not yet been fully established for the entire range of indications (dosage range, bolus: 10–100 mg/kg BW; infusion rate: 1–10 mg/kg BW/h). In principle, repeated administration of TXA appears to be more effective in pediatric heart surgery than a single bolus [106].

In a pharmacokinetic study of heart operations the recommended TXA dosage plan for children was a loading dose of 6.4 mg/kg BW with weight-adapted infusion rates ranging from 3.1 mg/kg BW/h to 2.0 mg/kg BW/h (BW 5–40 kg) [107].

Recent guidelines for the therapy of congenital platelet dysfunctions [108, 109] recommend for children the dosage plan summarized in Table 7.

**Table 7:** Dosing of tranexamic acid in children according to current guidelines (modified according to [108, 109])

Children and adolescents		Bolus	Maintenance dose from day 0
<50 kg	Oral	15–25 mg/kg on preceding evening or 1.5–2× dosage on day of surgery	15–25 mg/kg 3–4× daily
	Intravenous	10–15 mg/kg on day of surgery	10–15 mg/kg 3× daily
≥50 kg	Oral	1.0–1.5 g on preceding evening or 1.5–2× dosage on day of surgery	1.0–1.5 g 3–4× daily
	Intravenous	0.5–1.0 g on day of surgery	0.5–1.0 g 3× daily

The prescribing information on Cyklokapron® recommends different dosages for children [27]:

- oral: 15–25 mg/kg BW up to a maximum of 1.5 g 2–3× daily
- i. v.: For currently approved indications for children from the first year of life onwards 20 mg/kg BW/day; for these indications, only limited data on efficacy, dosages and safety are so far available.

Mouth washes (see footnote 3) with TXA are useful for the prevention and treatment of hemorrhage in the buccal mucosa. TXA can also be diluted in a drinkable liquid. If the liquid is swallowed after rinsing, the amount has to be allowed for in the overall dose [102].

### Epistaxis and tonsillectomy

Along with satisfactory results achieved by the topical (see footnote 3) application of TXA in the treatment of idiopathic epistaxis [110], a European randomized double-blind cross-over study (*n* = 118) found TXA (3 g/day) also to be effective in patients suffering from epistaxis associated with hereditary hemorrhagic telangiectasia in that the monthly duration of epistaxis was significantly shortened as compared with placebo (–17.3%, *p* = 0.0005) [111].

In the case of epistaxis TXA is at first applied topically to the nasal mucosa (moistened strip of gauze) by tamponading the nose cavum; if recurrent bleeding is expected the patient should be given 2 tablets (1 g) 3 times a day for 4–10 days before the tamponade is removed. In patients with a body weight less than 30–50 kg pediatric dose recommendations should be strictly adhered to.

A systematic review of older studies on the use of TXA with tonsillectomies indicated a significant reduction of blood loss as compared with the control groups, while TXA had no influence at all on the incidence of postoperative bleeding [112].

### Neurosurgical interventions and subarachnoid hemorrhage

In the course of neurosurgical interventions TXA (1 g immediately after diagnosis of an aneurysmal subarachnoid hemorrhage [aSAH], followed by 1 g every 6 h up to the time the aneurysm has been corrected) reduced the mortality risk due to early rebleeding by 80% [113]. Data from randomized controlled studies on the efficacy and safety of antifibrinolytics in brain surgery are rare and mostly older than the above cited Hillman study, or they relate to aprotinin, which was withdrawn from the market in 2007 [114]. A Cochrane review published in 2013 included all randomized controlled studies published between 1973 and 2002 [115]. The authors came to the conclusion that the short-term data were promising but felt that the studies were too heterogeneous to justify a general recommendation to use antifibrinolytics in the treatment of aSAB. Similarly, the European Stroke Organization is not ready to make such a recommendation on the basis of the data so far available [116].

The joint guidelines of the Austrian Neurological Society (Österreichische Gesellschaft für Neurologie, ÖGN) and the German Neurological Society (Deutsche Gesellschaft für Neurologie, DGN) published in 2008 explicitly do not recommend a prophylactic administration of antifibrinolytics for SAH (↓↓) [117], a position that was repeated in the updated version of 2012 [118].

The CRASH-2 intracranial bleeding study found that while patients with intracranial bleeding due to traumatic brain injury who were given TXA showed a significantly better outcome regarding the combined endpoint “poor outcome” (TXA vs placebo: 45% vs 58% of patients), the difference between TXA and placebo was not significant with regard to the individual parameters mortality (*p* = 0.06), recurring focal ischemia (*p* = 0.20), re-bleeding (*p* = 0.22) and substantial increase in bleeding (*p* = 0.13) [119].

Currently a number of studies are underway to evaluate the earliest possible short-term administration of TXA in patients with SAH (ULTRA), intracerebral bleeding (STOP-AUST) or traumatic brain injury (CRASH-3) [120–122].

## TXA not recommended by the authors

According to expert opinion, administration of TXA is not recommended in the following cases:

- Renal failure
- Epilepsy
- Benign gynecological interventions (e. g. myomectomy)
- In combination with activated factor concentrate (FEIBA, factor VIII inhibitor bypass activity; 1 ml = 25 E* factor VIII inhibitor bypass activity; see footnote 3)
- Fibrinolysis due to disseminated intravascular coagulation without any significant bleeding

If TXA were still to be administered, this should only be done in patients in whom an activation of the fibrinolytic system preponderates and in the presence of severe bleeding.

## Summary

TXA has been used for decades to prevent and treat clinically relevant HF and more generally for the prevention and treatment of bleeding due to a variety of causes. In the course of major surgical interventions (especially cardiac surgery, orthopedic surgery and liver transplantations) TXA reduces perioperative blood loss and the need for transfusions. In trauma patients with partly excessive bleeding TXA can also reduce mortality, in particular if administered within a narrow time frame after injury.

Despite extensive experience there still remain open questions as to the uses of TXA, which would ideally have to be dealt with in future studies:

- In some fields of application there is a need to define the optimum TXA dosage, since, side by side with the dose recommendations found in current summaries of product characteristics (SPC), both clinical studies and the practice of centres with considerable experience have in the meantime provided evidence that numerous other regimes, such as half doses, administration of a bolus, administration twice, preoperatively and postoperatively, seem to be equally effective.
- As to the risk of thromboembolic complications findings are still partly contradictory, and it is not yet clear which patients are at risk at all and which of them run the highest risk.
- This is also true of seizures observed with higher TXA doses and in the course of cardiac surgery.
- It would be highly desirable to develop better and more sensitive diagnostic methods to differentiate more easily between hyperfibrinolytic conditions and other coagulopathies or DIC. Likewise, suitable tests ought to be developed to identify more precisely patients that might be treated with TXA and those likely not to benefit from such treatment.

## Outlook

More extensive or more precise answers are expected from current randomized controlled studies comprising several thousands of patients, such as, for example, the Australian PATCH study (Pre-hospital Antifibrinolytics for Traumatic Coagulopathy and Haemorrhage – NCT02187120), which investigates early administration of TXA to severely injured patients to improve survival rates and convalescence, and the Danish PeTraH study (NCT01940419), which evaluates TXA administered to prevent bleeding in cases of benign hysterectomy. The French TRAAP study (Tranexamic Acid for Preventing Postpartum Haemorrhage Following a Vaginal Delivery – NCT02302456) investigates whether low-dose TXA immediately after vaginal delivery can reduce the incidence of postpartum hemorrhage in women having received oxytocin. Moreover, the international WOMAN trial (World Maternal Antifibrinolytic trial – NCT00872469), a randomized double-blind study, in which more than 20,000 women from 21 countries with postpartum bleeding are enrolled, evaluates the efficacy of early administration of TXA with regard to the endpoints death, hysterectomy, surgical interventions, blood transfusions and other vascular events (http://www.womantrial.lshtm.ac.uk/).

## Footnotes

1
http://www.who.int/selection_medicines/committees/expert/18/applications/tranexamic/en/.

2 Therapeutic recommendations are made on the basis of evidence classes. Recommendation grade A (= ‘is to’): at least one randomized controlled study of overall good quality and consistency, which is directly related to the recommendation in question and has not been extrapolated (evidence levels Ia and Ib [34]).

3 Topical administration is not mentioned in the prescribing information.

4
*H*emorrhage *AL*leviation with *T*ranexamic acid – *I*n*T*estinal system; the study protocol is available on the Internet: http://haltit.lshtm.ac.uk/.
